# The Microcalorimeter for Industrial Applications

**DOI:** 10.6028/jres.107.050

**Published:** 2002-12-01

**Authors:** Del Redfern, Joe Nicolosi, Jens Höhne, Rainer Weiland, Birgit Simmnacher, Christian Hollerich

**Affiliations:** EDAX Inc., 91 McKee Dr., Mahwah, NJ 07430 USA; CSP Cryogenic Spectrometers GmbH, Munich, Germany; Infineon Technologies AG, Munich, Germany; Technische Universita¨t München, Munich, Germany

**Keywords:** cryogen-free cooling, energy resolution, microcalorimeter energy dispersive, transition-edge sensor, x-ray microanalysis, x-ray spectrometer

## Abstract

To achieve the dramatic increases in x-ray spectral resolution (<20 eV at 1.5k eV) desired by market segments such as the semiconductor industry, NIST developed a transition-edge sensor (TES) microcalorimeter. To bring this exciting, yet demanding, new technology to the industrial users, certain criteria must be addressed. Aspects of resolution, cooling and hold time, count rates as well as vibrations are considered. Data is presented to the present efforts to handle these issues as well as discussing development plans for the future.

## 1. Introduction

The requirement for improvement in x-ray detector technology has been a major necessity for the semiconductor industry in their long term goal to address the analytical requirements of particles down to 35 nm as discussed in the 1997 National Technology Roadmap for Semiconductors (NTRS) [1]. To achieve these analytical requirements on the scanning electron microscope, low excitation voltages must be used, and therefore low energy x-ray spectrometry is a necessity. An increase in resolution of the energy dispersive x-ray spectrometer by an order of magnitude is required. To achieve this dramatic increase in resolution (<20 eV at 1.5 k eV), NIST developed a transition-edge sensor (TES) microcalorimeter [2]. The NIST energy dispersive microcalorimeter spectrometer for x-ray microanalysis used cryogens and an adiabatic demagnetization refrigerator (ADR) to achieve the working temperatures (<100 mK) of the superconducting TES.

To bring this exciting technology to industrial applications, EDAX INC^1^ and CSP (Cryogenic Spectrometers) GmbH established a partnership to develop a commercially available microcalorimeter and installed the first beta unit at Infineon Munich.

## 2. Requirements for Industrial Applications

To move from a research tool to a commercially accepted routine analytical tool there are certain criteria that need to be met. These criteria include energy resolution, cooling and available hold time, count rates, and vibration. Development work has been completed (and further developments planned) on each criterion to achieve the requirements for industrial applications.

### 2.1 Resolution

The semiconductor industry has a requirement for small particle (<100 nm) analysis to determine their composition [1]. Typical applications within the industry [3] are to establish the simultaneous presence of Ti and N, Si, and W, or Si and Ta in particles with nanoscale dimensions. To achieve these requirements, low excitation voltages have to be applied, thus forcing the analyst to use low energy x-ray spectra to confirm the presence of the elements of interest. Therefore the first criterion an energy dispersive microcalorimeter x-ray spectrometer must be able to achieve is a resolution of <20 eV at 1.5k eV.

**Fig. 1:**
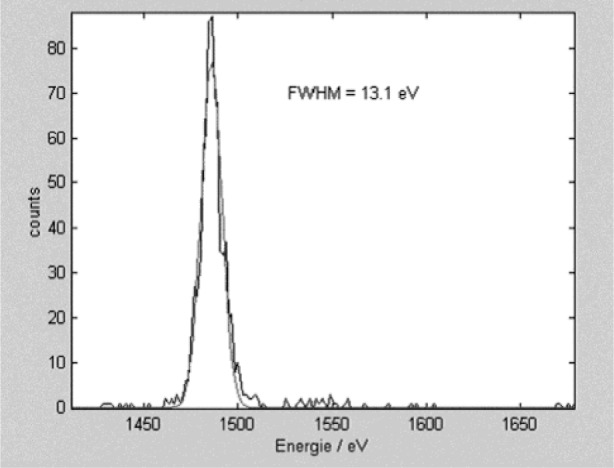
Aluminum spectrum with 13 eV resolution (smooth trace = fitted peak).

**Fig. 2:**
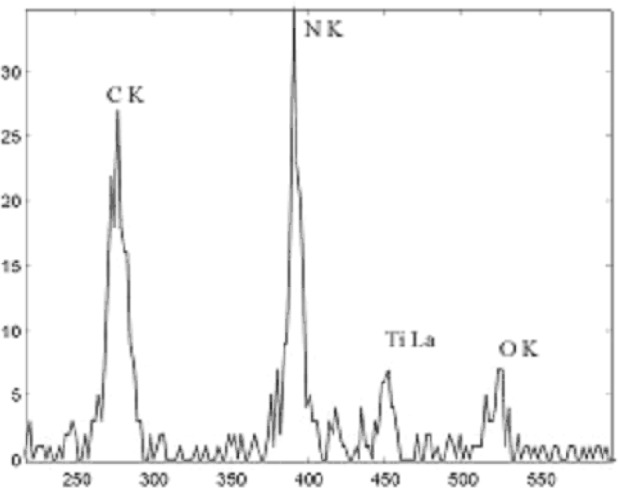
Ti-N spectrum with C and O present.

**Fig. 3:**
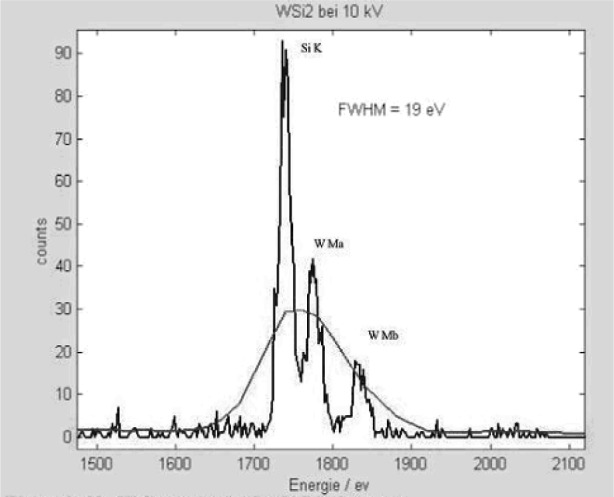
Si-W spectrum (smooth trace = fitted peak).

**Fig. 4:**
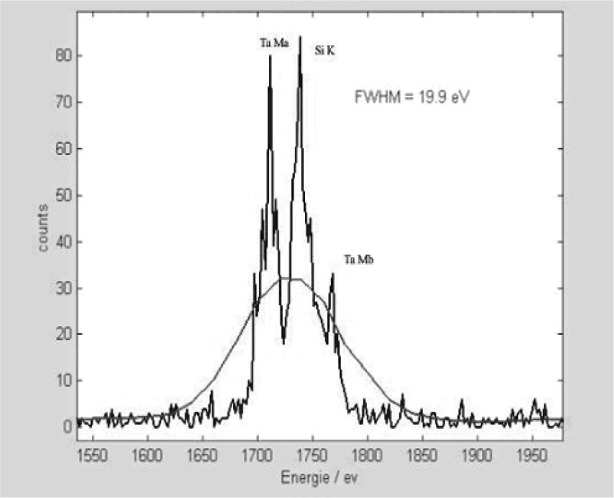
Si-Ta spectrum (smooth line = conventional Si(Li) EDS).

Figure 1 shows a spectrum of Al measured on the microcalorimeter with an energy resolution of 13 eV. Figures 2, 3, and 4 demonstrate the ability of the microcalorimeter to resolve important peak interference problems: N K and Ti Lα (Fig. 2), Si K from W M (Fig. 3) and Si K from Ta M (Fig. 4). The red lines in the spectra are comparisons against a typical Si(Li) energy dispersive detector.

### 2.2 Cooling and Hold Time

**Fig. 5:**
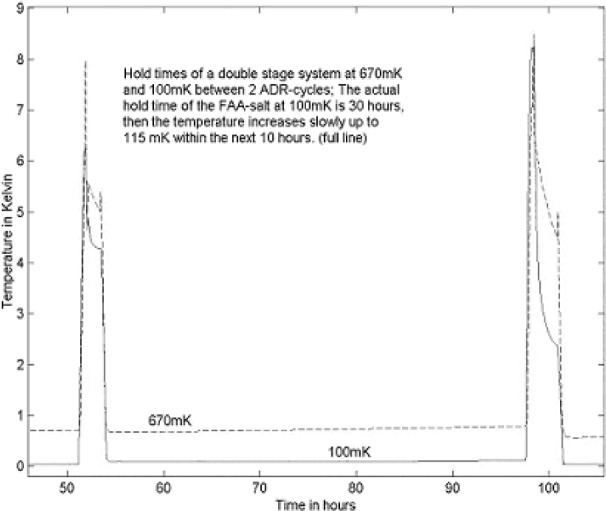
Cooling curve with extended hold time unit.

The second criterion is cooling. The NIST microcalorimeter employed liquid nitrogen and liquid helium to achieve a temperature of 4 K. An ADR [4] was then used to take the temperature down to the operating range of the TES of <100 mK. To bring the microcalorimeter into a semiconductor manufacturing environment where the use of liquid cryogens is discouraged, CSP developed the pulse tube cooling system, a mechanically cooled device that will generate temperatures to <4 K without the use of cryogens. The CSP microcalorimeter employs the ADR in the final cooling stage to reach temperatures of <100 mK. The ADR [4] has a working period at low temperature of approximately 10 h. After this time the ADR has to be recharged (this procedure takes approximately 1 h) and then it is available for further use. A major requirement for the industrial tool is automation and longer available working time of the system. The mechanically cooled microcalorimeter can be programmed to begin the recharging cycle of the ADR at a pre-programmed time. The working time of the salt pill can be extended to over 24 h by employing the “extended hold time” (EHT) unit. The use of the “extended hold time” unit enables the analyst to have “full” availability of the microcalorimeter should they find any one hour free during the day. Figure 5 shows the ADR working temperature with the EHT unit employed with a working temperature of 30 h.

### 2.3 Count Rates

The acquisition times required by the semiconductor industry are <60 s per particle. The detector (absorber) area is only 400 μm^2^ at a working distance of 15 mm. The typical counts per second achievable using the present configuration of the microcalorimeter are around 6 counts per second (using 5kV, 700 pA). This may seem very low but considering the background levels are extremely low and the resolution of the detector very high most of the counts collected are very meaningful. A typical acquisition time is around 3 min. Within 1 min an experienced operator can determine the presence of the elements of interest, but 3 min of spectral accumulation is required for 100 % confidence.

To achieve the required count per seconds there are four areas that can be evaluated. The first is the simplest, which is to match the collimator with the detector size. The present system has a circular collimator and a square detector, and these will be matched in the future. The second modification will be to improve the solid angle of the detector, which at present is not optimum. These two minor changes to the configuration are expected to increase the count rate by a factor of 6. This improvement should bring the acquisition time below 60 s, but two other modifications are possible. The first is the use of polycapillary optics [5]. These optics utilize high-efficiency grazing-angle x-ray reflections to deflect x rays over a wide angle. Using these optics an increase in counts per second of over 10 fold should be possible. The last option to increase the counts per second is to use a larger-area absorber (this is a long term development plan and will not be considered for this generation of microcalorimeter).

### 2.4 Vibration

**Fig. 6:**
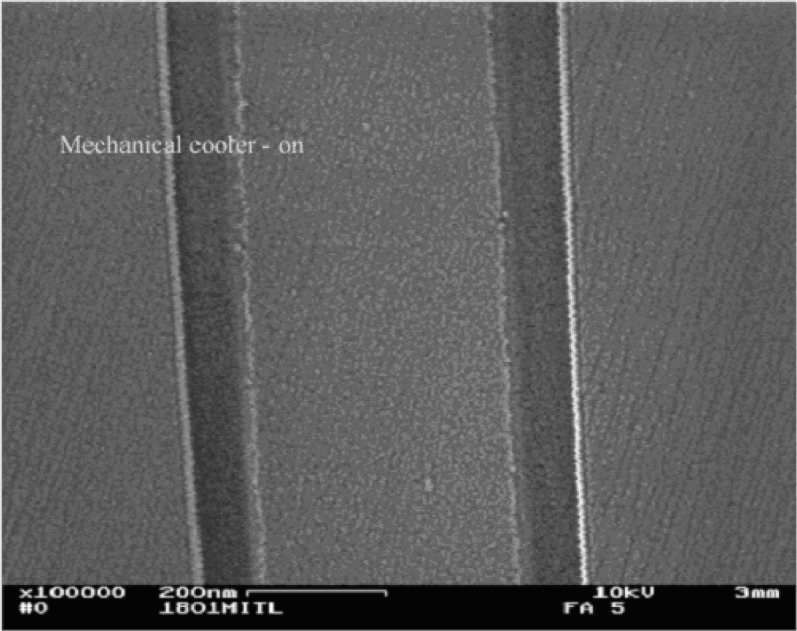
SEM image at 100 000× magnification with cooler on. Note vibration effects at bright edges and loss of fine-scale details.

**Fig. 7:**
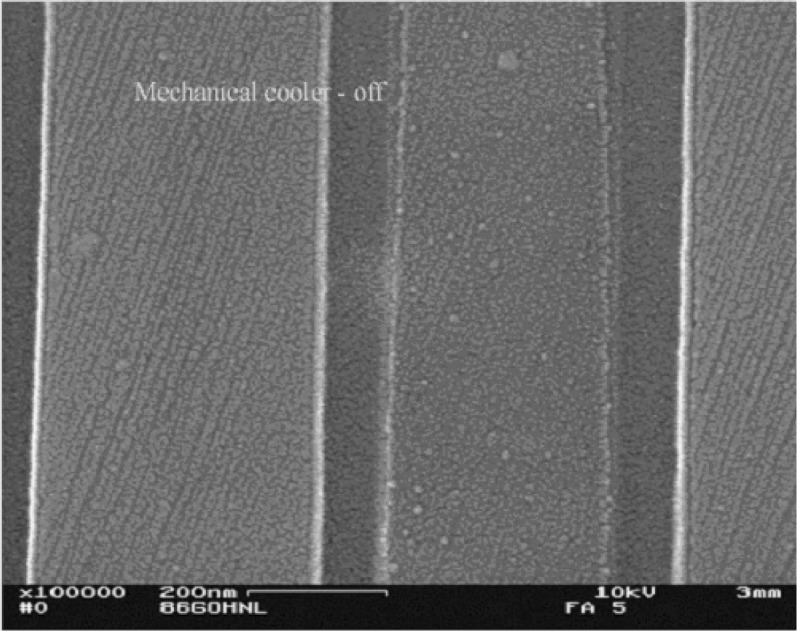
SEM image at 100 000× magnification with cooler off. Note improved edge image and recovery of fine-scale details.

A high resolution SEM has strict criteria for vibration requirements. The CSP / EDAX Polaris microcalorimeter energy dispersive x-ray spectrometer weighs approximately 50 kg and is mounted to the chamber via a port adapter. The only moving part of the microcalorimeter is the rotating valve that stands on top of a pole and is connected to the system via thin tubing. Vibration measurements have been taken, and Figs. 6 and 7 show the effect of the mechanical cooling on the SEM of an image taken at 100 000 magnification. The image with the cooler on shows a slight effect of vibration.

**Fig. 8:**
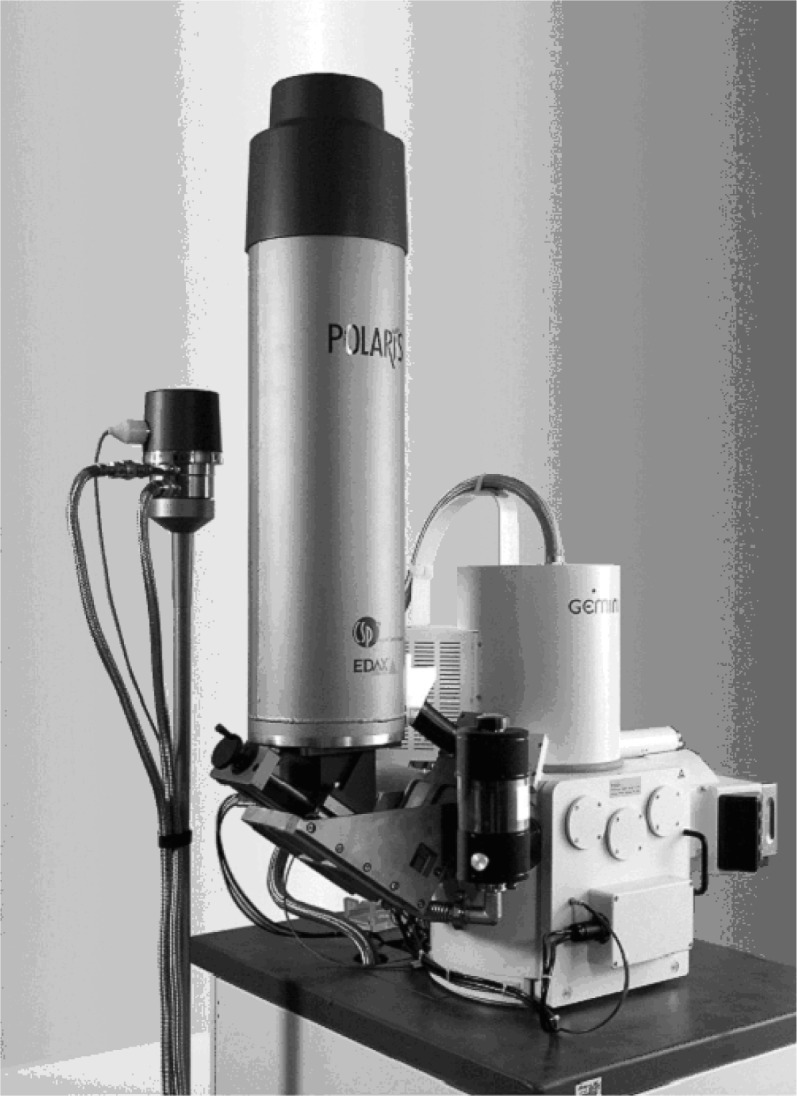
Polaris microcalorimeter energy dispersive x-ray spectrometer.

The last issue to resolve for the microcalorimeter energy dispersive x-ray spectrometer to be completely acceptable for industrial applications is a noise issue. The rotating valve (located on the pole at the side of the Polaris system shown in Fig. 8) produces a pinging sound at low frequency which will be reduced to background values with appropriate shielding.

## 3. Conclusion

Today the microcalorimeter energy dispersive x-ray spectrometer installed at Infineon, Munich is able to acquire a spectra with a resolution of 15 eV at 1.5k eV within 3 min, has a hold time of 8 h, and is mechanically cooled. There are plans to further develop the system to ensure it satisfies all criteria for the industrial applications. These plans are designed to increase the count rate of the beta unit and include modifications to the geometry, i.e., the collimator and solid angle, testing of the polycapillary optics and alig nment issues, and larger-area x-ray absorbers. The EHT unit will enable the microcalorimeter to be available for periods >24 h. Vibration issues still have to be resolved to ensure vibration free operation at magnifications of 200 000×. For example, the noise of the rotating valve has to be reduced to background levels. Once all these modifications and improvements have been completed, we will see the microcalorimeter energy dispersive x-ray spectrometer commercially available for industrial applications.

## Footnotes

1 NIST disclaimer: Certain commercial equipment, instruments, or materials are identified in this paper to foster understanding. Such identification does not imply recommendation or endorsement by the National Institute of Standards and Technology, nor does it imply that the materials or equipment are necessarily the best available for the purpose.
